# Comparative Study of Cyanobacterial and *E. coli* RNA Polymerases: Misincorporation, Abortive Transcription, and Dependence on Divalent Cations

**DOI:** 10.4061/2011/572689

**Published:** 2011-10-12

**Authors:** Masahiko Imashimizu, Kan Tanaka, Nobuo Shimamoto

**Affiliations:** ^1^Structural Biology Center, National Institute of Genetics, and Department of Genetics, The Graduate University for Advanced Studies, Mishima, Shizuoka 411-8540, Japan; ^2^Gene Regulation and Chromosome Biology Laboratory, National Cancer Institute at Frederick, MD 21702, USA; ^3^Graduate School of Horticulture, Chiba University, 648 Matsudo, Matsudo-shi, Chiba 271-8518, Japan; ^4^Faculty of Life Sciences, Kyoto Sangyo University, Kamigamo-Motoyama, Kita-Ku, Kyoto 603-8555, Japan

## Abstract

If Mg^2+^ ion is replaced by Mn^2+^ ion, RNA polymerase tends to misincorporate noncognate nucleotide, which is thought to be one of the reasons for the toxicity of Mn^2+^ ion. Therefore, most cells have Mn^2+^ ion at low intracellular concentrations, but cyanobacteria need the ion at a millimolar concentration to maintain photosynthetic machinery. To analyse the mechanism for resistance against the abundant Mn^2+^ ion, we compared the properties of cyanobacterial and *E. coli* RNA polymerases. The cyanobacterial enzyme showed a lower level of abortive transcription and less misincorporation than the *E. coli* enzyme. Moreover, the cyanobacterial enzyme showed a slower rate of the whole elongation by an order of magnitude, paused more frequently, and cleaved its transcript faster in the absence of NTPs. In conclusion, cyanobacterial RNA polymerase maintains the fidelity of transcription against Mn^2+^ ion by deliberate incorporation of a nucleotide at the cost of the elongation rate. The cyanobacterial and the *E. coli* enzymes showed different sensitivities to Mg^2+^ ion, and the physiological role of the difference is also discussed.

## 1. Introduction

A DNA-dependent RNA polymerase (RNAP) has a catalytic center chelating Mg^2+^ ions to form phosphodiester bonds [1, 2]. Most other divalent cations inhibit the activity, but Mn^2+^, which has a similar but only slightly larger ionic radius than Mg^2+^, supports the polymerizations. In the case of *Escherichia coli* (*Esc*) RNAP, Mn^2+^ causes the enzyme to misincorporate deoxyribonucleoside triphosphate (dNTP) instead of ribonucleoside triphosphate (NTP) [3] as well as noncognate NTPs [4–6]. Therefore, the growth of *E. coli* is inhibited in the presence of Mn^2+^, and the inhibition is mitigated by GreA and GreB which bind to RNAP [7].

In cyanobacteria, the intracellular concentration of Mn^2+^ is considered to be higher by two orders of magnitude than in *E. coli* [8], because Mn^2+^ is required at higher concentrations to assemble the photosynthetic reaction center carrying Mn^2+^ cluster, and/or to scavenge reactive oxygen species generated from photosynthesis [9, 10]. Since cyanobacteria have no genes orthologous to *greA* or *greB*, cyanobacteria should have the mechanism of detoxifying Mn^2+^. 

We thus made two purified reconstituted transcription systems from a mesophilic cyanobacterial species, *Synechococcus* sp. PCC 7942 (*Syn*), and from a moderately thermophilic species, *Thermosynechococcus elongatus* BP-1 (*The*). By comparing these systems with the system of *E. coli*, we here examine the possibility that the mechanism of detoxifying Mn^2+^ is installed in the poisoning target, RNAP, and ask whether or not the cyanobacterial RNAP has a sensitivity to Mg^2+^ different from that of *E. coli* RNAP. 

## 2. Materials and Methods

### 2.1. Materials

All of the oligo DNAs and RNA, NTPs, and [*γ*-^32^P]ATP were obtained from Hokkaido System Science, Yamasa and Perkin Elmer, respectively. Restriction enzymes were purchased from New England Biolabs and Takara. Primestar HS and Primestar Max DNA polymerases used for PCR were purchased from Takara. *Syn* cells used for RNAP purification were partially gifted from Dr. Mitsumasa Hanaoka. The culture condition of the gifted cells is the same as shown in the next section.

### 2.2. Protein Purification

Cells of *Syn* and *The *were cultivated and harvested as described [15, 16]. About 10 g of the wet-cell paste was suspended in 30 mL of TGED buffer [10 mM Tris-HCl (pH 8.0), 5% (v/v) glycerol, 0.1 mM EDTA, and 1 mM DTT] containing 1 mM phenylmethylsulfonyl fluoride and 0.2 M NaCl, and then, cells in suspension were lysed by sonication. A 35%- saturated solution of ammonium sulfate was added to a soluble fraction of the cell lysate which was obtained by centrifuging for 30 min at 20,000 × g. The obtained mixture was added to 10 mL of phenyl sepharose resin (GE Healthcare), pre-equilibrated with the TGED buffer containing 0.2 M NaCl and 35% saturated ammonium sulfate, and the mixture was washed by the same buffer, eluted with the TGED buffer. The eluate was applied to a column containing 10 mL of DEAE resin (TOSOH) pre-equilibrated with the TGED buffer, and the column was washed with TGED buffer containing 0.2 M NaCl, eluted with the TGED buffer containing 0.5 M NaCl. The eluted fraction was further chromatographically purified by using Hi-trap heparin affinity column (GE Healthcare) and MonoQ anion exchange column (GE Healthcare) as described [16]. The purified core enzyme was dialyzed against the TGED buffer containing 0.2 M NaCl and 50% glycerol and stored at −80°C. The core enzyme of *E. coli* was prepared according to [17], and *σ* ^70^ of *E. coli* and **σ*^A^*s of *Syn* and *The* were obtained as described in [15, 16].

### 2.3. *In Vitro* Transcription Assays

The liner DNA template containing the T7A1 promoter from −147 to +87, when +1 is the transcription start site, was prepared by PCR using the plasmid pAR1435 [18] and the following digestion by *Hae*III. The template containing the *psbA2* promoter from −127 to +101 was prepared by PCR using a genomic DNA of a cyanobacterium *Synechocystis sp*. PCC 6803. These DNA templates were purified by PAGE. The holoenzyme was reconstituted by incubating core RNAP mixed with a 3-fold molar excess of the primary *σ* factor for 10 min at 37°C. We used twice more *Syn* and *The* RNAPs, because they have smaller affinities for the *psbA2* promoter. The reconstituted holoenzyme (50 nM for *E. coli* or 100 nM for *Syn* and *The*) and 20 nM DNA template was preincubated for 10 min at 37°C in 8 *μ*L of T-buffer [50 mM Tris-HCl (pH 7.9), 100 mM KCl, 10 mM MgCl_2_ (see figure legends and the main text in the cases that the concentration was changed or MgCl_2_ was changed to MnCl_2_), 1 mM DTT, and 150 *μ*g mL^−1^ partially hydrolysed casein]. Reaction was started by adding 2 *μ*L of prewarmed substrate mixture: 5 *μ*M [*γ*-^32^P]ATP (40 Ci mmol^−1^) as well as 100 *μ*M each of GTP, CTP, and UTP. In a single-round reaction, heparin (100 *μ*g mL^−1^) was added together with the substrates to eliminate enzyme turnover. After incubation for 20 min at 37°C, the reaction was stopped by phenol/chloroform/isoamyl alcohol (25 : 24 : 1). Transcripts were analyzed by PAGE using a 20% gel containing 7 M urea. The sequences of abortive transcripts from the T7A1 promoter were assigned according to [19], and those from the *psbA2* promoter were determined using 5′ end-labeled RNA marker 5′-AGUCAGUU-3′ and a chemical cleavage assay (Figure  S2 which is available online at doi:10.4061/2012/572689). The assignment of abortive transcripts are often confused by the presence of cleaved fragments of the run-off transcript. The cleavage occurs during the extraction with oxidized phenol but the fragments are distinguished by the retention of 3′-phosphate as described in Figure S2 which is available online at doi:10.4061/2012/572689.

All the experiments with presented results in this study were repeated two or more times and the represented ones are shown. 

### 2.4. TEC Formation

In order to stop elongation at Position +18 by a lack of the cognate CTP for Position +19, the intrinsic cytosine residues (nontemplate strand) at Positions +4, +9, +10, and +14 of the *psbA2* promoter were replaced by thymine by PCR-based mutagenesis, following cloning of the DNA template from −127 to +101 into a plasmid pUC19 (see Figure 4(a)). For the TEC9 formation, the intrinsic cytosine residues at Positions +10 and +14 were retained. The binary complex was formed by mixing the holoenzyme (50 nM for *E. coli* or 100 nM for *Syn*) and 40 nM DNA template containing the* psbA2* promoter as described above. The ternary complex was formed by incubating 5 min at 37°C with 5 *μ*M [*γ*-^32^P]ATP (40 Ci mmol^−1^) as well as 50 *μ*M each of GTP and UTP in T-buffer containing 10 mM MgCl_2_ or 1 mM MnCl_2_. The TEC was isolated from the substrates including [*γ*-^32^P]ATP as well as released abortive transcripts by triplicated passing through a MicroSpin G50 column (GE Healthcare) equilibrated with T2-buffer [50 mM Tris-HCl (pH 7.9), 100 mM KCl, 10 mM MgCl_2_ or 1 mM MnCl_2_, 1 mM DTT, and 5% glycerol] at room temperature. After this treatment, no elongations by the TEC9s and TEC18s of both *Syn* and *E. coli* in the T2 buffer were detected for 10 min at 37°C, without adding substrates. 

### 2.5. Single-Step Elongation Assay

Reactions were started by adding cognate or noncognate NTP at the final concentration of 0.5 mM to 9 *μ*L solution containing the TEC and the T2-buffer. The obtained mixture was incubated at 37°C for the indicated time and the reaction was stopped by adding an equal volume of gel-loading solution, containing 95% (v/v) deionized formamide, 20 mM EDTA (pH 8.0), bromophenol blue and xylene cyanol, 0.05% (w/v) each, and analyzed by PAGE using a 20% gel containing 7 M urea.

## 3. Results

### 3.1. Cyanobacterial Transcription System Reconstituted from Purified Components

**Figure 1:**
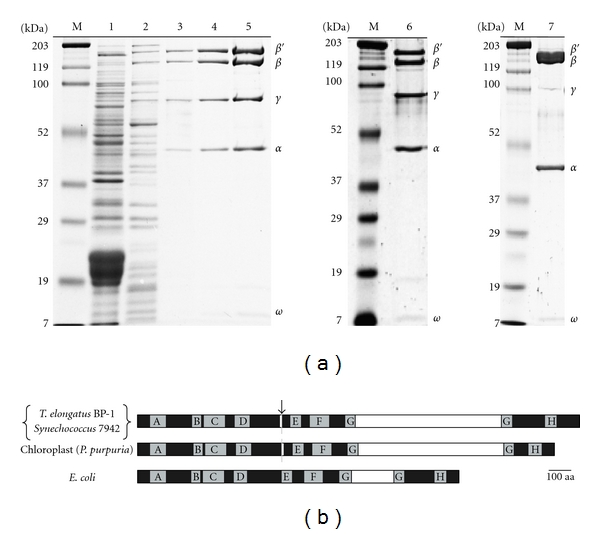
Cyanobacterial RNAPs. (a) Analysis of the fractions of *The *core enzyme by 10% SDS-PAGE after the treatment of hydrophobic resin (lane 1 : 26.9 *μ*g), DEAE anion-exchange (lane 2 : 7.0 *μ*g), heparin affinity (lane 3 : 0.8 *μ*g), and MonoQ chromatography (lane 4 : 1.6 *μ*g). Lanes 5–7 show 7.5 *μ*g of the purified *The*, *Syn, *and *E. coli *enzymes used in transcription assays. Marker proteins (BIO-RAD) are also shown in lane M with the molecular weights indicated in the left margin. The gel was stained with Coomasie Brriliant Blue. (b) Schematic diagrams of *β*′ subunits of *The*, *Syn*, a chloroplast of red alga *Porphyra purpurea, *and *E. coli*. The conserved regions A–H [11, 12] were also indicated. The white boxes represent the nonconserved domain inserted in Region G [13, 14]. The split sites are indicated by an arrow. The scale bar of 100 amino-acid residues (aa) is indicated at the bottom.

For the comparative study, it is essential to purify cyanobacterial RNAPs. The tedious step of the purification is to remove thylakoid-membrane fragments and the associated proteins [20, 21]. We thus invented a batch-wise removal with a hydrophobic resin (see Materials and methods). The *Syn* and *The* core enzymes were purified by the improved procedure, resulting in a preparation which is more than 95% pure, judged by CBB stain, within a day (Figure 1(a)). The core enzyme of cyanobacteria is composed of *α*_2_ *ββ*′*ω* as *E. coli* enzyme, but its *β*′ subunit is composed of two polypeptides [20]. A large nonconserved domain is inserted in the G region as chloroplast RNAP (Figure 1(b)) [21].

**Figure 2:**
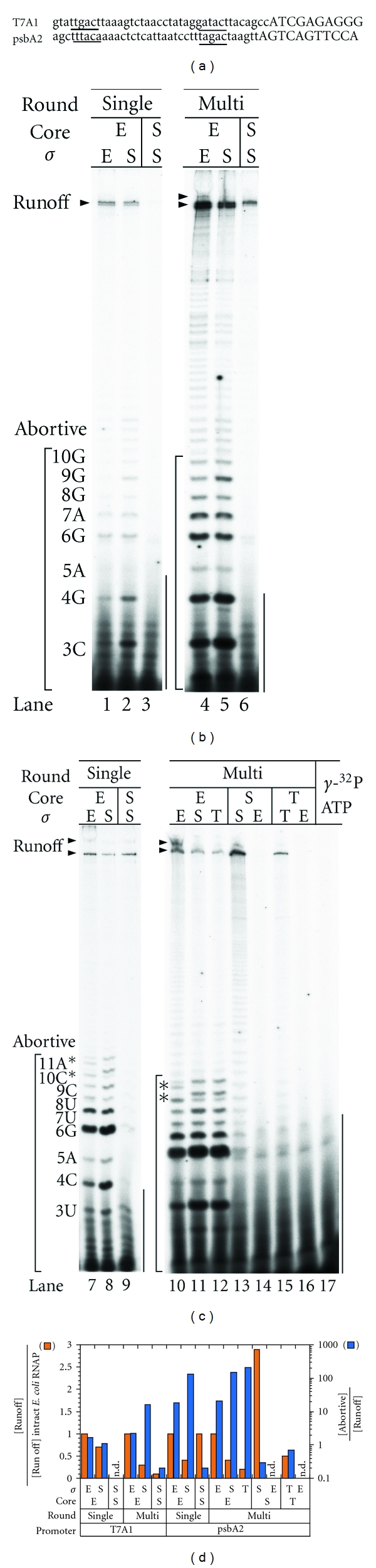
Transcription from the T7A1 and the *psbA2 *promoters by cyanobacterial and *E. coli *RNAPs as well as their chimeric enzymes. (a) The DNA sequences of the T7A1 and the *psbA2 *promoters. The putative −10 and −35 elements are underlined, and the transcribed sequences are indicated in uppercase letters. (b) The analysis of transcripts labeled with [*γ*-^32^P]ATP from the T7A1 promoter with 20% polyacrylamide in the presence of 7M urea in TBE buffer. (c) The transcripts from the *psbA2 *promoter. The round of transcription is indicated at the top of the gels. The runoff and abortive transcripts are indicated by arrowheads and parentheses, respectively. The length and the incorporated nucleotide at its 3′ end of an abortive transcript are indicated on the left margin. Asterisks indicate the abortive transcripts involving misincorporation. The radioactive contaminants that is contained in [*γ*-^32^P]ATP are also indicated in lane 17. (d) The amounts of the run-off transcript normalized by that of the intact *E. coli *enzymes (red) and the ratio of the amount of abortive transcripts to that of the run-off transcript in logarithmic scale.

We examined the transcription by the holoenzymes of *Syn*, *The,* and *E. coli* retaining their primary *σ* factors (*σ* ^s^ and *σ* ^70^) at the T7A1 promoter (Figure 2(a)), which is the strongest among the standard promoters for the *E. coli* enzyme [22]. The *Syn* enzyme was active on this transcription unit but much weaker than the *E. coli* enzyme: the runoff transcript was observed only at the multiround condition, and its amount was lower by an order of magnitude (Figure 2(b)). Moreover, the *The* enzyme did not synthesize any detectable transcripts in all conditions (data not shown).

We thus searched for a promoter driving transcription by both cyanobacteria and *E. coli* holoenzymes without adding specific activators. The cyanobacterial *psbA2* gene encoding D1 protein of the photosystem II is highly transcribed in the daytime [23] and the sequence of its promoter shares the −10 and −35 elements recognized by the *E. coliσ* ^70^ holoenzyme (Figure 2(a)) [24]. As expected, the *E. coli* enzyme was active on this promoter and produced similar amounts of runoff transcripts from the *psbA2* promoter in the single-round condition (Figure 2(c)). We thus used this promoter in comparison between these enzymes.

### 3.2. Cyanobacterial RNAPs Are Less Abortive due to Their Core Parts

In contrast to the similar amounts of runoff transcript, *Syn* RNAP produced a much lower level of 2–11 nt long transcripts (Figure 2(c)), which is known as abortive transcription, an iterative synthesis and release of oligo-RNA in initiation [25, 26]. The ratio of abortive synthesis to runoff synthesis is smaller than that in *E. coli* by two orders of magnitude. The ratio for *The* RNAP could be determined only in a multiround transcription condition and was as low as that of *Syn* RNAP (Figure 2(c)). 

 The observed small ratios were not specific to the *psbA2* promoter, because in multiround transcriptions from the T7A1 promoter, the ratio for *Syn* RNAP was also two orders of magnitude smaller than that for *E. coli *(lanes 4 and 6 in Figure 2). In this experiment, we used the holoenzymes reconstituted from the **σ*^A^*s purified from the overproducing *E. coli *strains because of low yields of holoenzymes. The observed low activities of abortive transcription are not due to the artifacts of the reconstitution. We independently purified the histidine-tagged holoenzyme of a *Synechocystis* sp. PCC 6803 and it also produced abortive transcripts as low as the *Syn *and *The *RNAP (Figure  S1A). In conclusion, cyanobacterial RNAPs are generally much less abortive than *E. coli* RNAP.

The level of abortive transcription is known to depend on mutations of both core enzyme and *σ* factor [27–29], indicating that both the components are responsible. We addressed the question of which component is more responsible by constructing chimeric holoenzymes. Taking into account the large difference between the levels of cyanobacterial and *E. coli *RNAPs, we found that the large difference was associated with the core part of RNAP but not *σ* (lanes 2, 5, 8, 11, and 12 in Figure 2). In spite of the absence of detectable transcripts by *The* enzyme in Figure 2, the above conclusion is also applicable to the enzyme because of the results of transcripts labeled with [*α*-^32^P] UTP (Figure  S1B). 

Among the abortive transcripts at the *psbA2* promoter, the 10 nt- and 11 nt-transcripts of *E. coli* RNAP accompanied the bands with decreased migration (Figure 2(c)). These transcripts were shown to contain a purine nucleotide instead of the cognate cytosine at Position +10, a misincorporation, by a chemical cleavage assay (Figure  S2). In contrast to abortive transcripts, the runoff transcript did not contain any detectable misincorporation. The chimeric enzymes containing *E. coli* core enzyme also showed the misincorporation (lanes 7, 8, 10–12 in Figure 2). Such a misincorporation by the *E. coli* enzyme had already been known for several promoters [19], and thus, this result suggests that the core enzyme is responsible for the misincorporation. 

When the enzymes of two different species with different typical growing temperatures are compared, there are no absolute choice of the temperature. Since we focused to the difference in the catalytic properties of RNAPs rather than the difference in its role in their growth, we selected the same 37°C for *in vitro* assays. In this way, we can avoid the effect of the different stabilities of DNA duplex in the promoter. The both cyanobacteria grow and survive at 37°C although the optimal temperature of *The *is 55°C. Another lines of circumstantial evidence for our choice is that the levels of the runoff RNA synthesis are similar for the *The* and *E. coli* enzymes for the *Syn rrnA* promoter (Panel B in Figure  S1).

### 3.3. *Syn* RNAP Is Likely to Form Moribund Complex which Produces Only Abortive Transcripts

**Figure 3:**
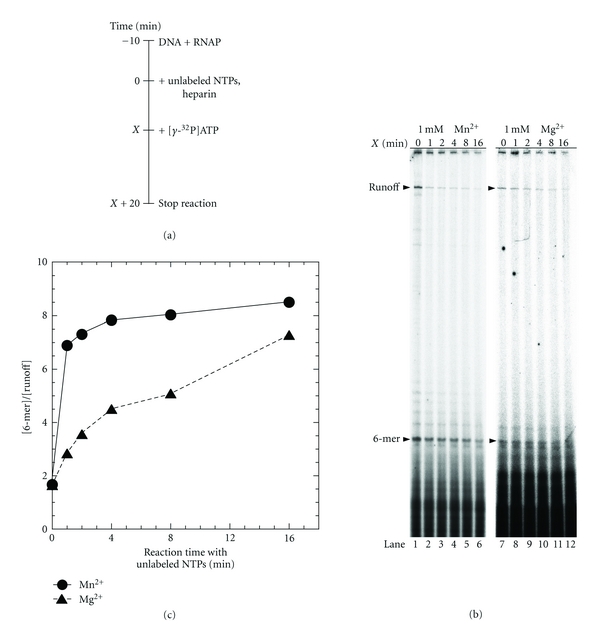
Inverse pulse-labeling assay for detecting persistent abortive synthesis at the *psbA2 *promoter by *Syn *RNAP. (a) Schematic diagram of the assay. The reaction time *X* is varied. (b) The labeled products synthesized at 1 mM Mn^2+^ (left) and at 1 mM Mg^2+^ (right). The runoff transcript and the 6-mer abortive transcript are indicated by arrowheads. (c) The ratios of the amount of 6-mer abortive transcript to that of the runoff transcript were plotted against the reaction time *X*.

For *E. coli* RNAP, only a part of the promoter complex synthesizes the full-length RNA, while the rest produces the majority of abortive transcripts at the promoter (Figure  S3), the latter being named moribund complex [7, 30, 31]. To test whether or not the *Syn* RNAP has a similar property, we carried out the most sensitive assay for detecting the moribund complex: inverse pulse-labeling which monitors the fates of promoter-RNAP complex [30]. In this assay, a single-round transcription from the *psbA2* promoter was started with unlabelled 4NTPs (ATP, CTP, GTP, and UTP) at time zero. The *γ*-^32^P-labeled initiating ATP was then added at various time points, followed by incubating for a further 20 min to complete the round (Figure 3(a)). Since the [*γ*-^32^P]-labeled runoff and abortive transcripts are both produced only by the RNAP that still survive at the promoter at the time point of adding [*γ*-^32^P]ATP, the ratio of the two kinds of labeled transcripts should reflect the preference of producing abortive transcripts. 

As shown in Figures 3(b) and 3(c), the preference becomes stronger at the later time points, showing that the RNAP bound at the promoter at time zero is not homogeneous. Although there is no evidence that cyanobacterial RNAP forms the same moribund complex as *E. coli *RNAP, which produces only abortive products, a fraction of the *Syn* enzyme produces abortive transcripts preferentially over others, and the fraction is enriched at the later time points.

### 3.4. *Syn* RNAP Pauses More Frequently and Elongates Transcript More Slowly Than the *E. coli* Enzyme

**Figure 4:**
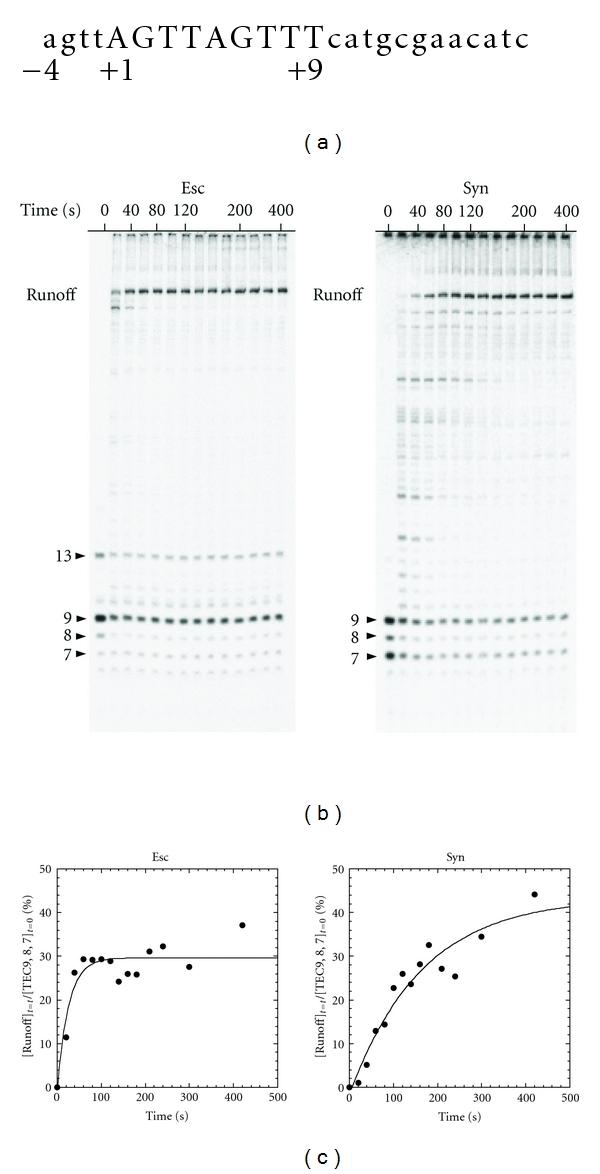
Elongation by the *Syn *and *E. coli *TEC9s formed at the *psbA2 *promoter. (a) A sequence of the nontemplate strand near the initiation site (+1). The early transcribed region of 9 mer is shown in uppercase letters and the upstream sequence in lower case letters. (b) The transcripts obtained in elongation of the TEC9s which had been labeled with [*γ*-^32^P]ATP. Four NTPs of 100 *μ*M each were added to the TEC9 at time zero and incubated for the indicated times. Only this experiment was performed at 25°C because of distinct pausing of *Syn *enzyme. The transcripts existed at time zero as well as the runoff transcripts are indicated by arrowheads. (c) The amount of the runoff transcript at each time point is normalized with the initial amount of TECs and plotted against time. The curves were the best-fit single-exponential ones.

We next compared the elongation by *Syn* with that by *E. coli* RNAPs. To isolate elongation from initiation, we used a ternary elongation complex retaining 9-mer transcripts, TEC9 (Figure 4(a)). It was elongated with 4NTPs in the presence of 10 mM Mg^2+^. The *E. coli* TEC9 smoothly elongated its transcript, and the elongation was completed at 40 s (Figure 4(b)). The 6- and 7-mer transcripts existed at time zero, and their amounts did not change, demonstrating that they had been contaminating TEC9. The 9-mer transcript, as well as a part of the 8-mer transcript, was elongated. Therefore, there is no significant pausing of the *E. coli *enzyme on this template.

 In contrast, the elongation of the *Syn* TEC9 was paused at a number of lengths and was completed at later than 200 s (Figure 4(b)). When the time courses of syntheses of the run-off transcripts are approximated to single-exponential curves, the elongation of the *Syn* TEC9 has a time constant of 6 times longer than that of the *E. coli *TEC9, that is, time constants of 164 s compared with 26 s (Figure 4(c)). 

The *Syn* TEC9 contains more amounts of 7-mer and 8-mer transcripts than the *E. coli* TEC9 at time zero. Since no such short transcripts were found prior to the step of removing NTPs in the preparation (data not shown), the shorter transcript must be generated in the absence of NTP by hydrolysis and/or pyrophosphorolysis of TEC9's, suggesting that the* Syn* TEC9 had a higher activity of shortening a transcript than *E. coli* had. In addition, the *E. coli *TEC9 is contaminated by a 13-mer transcript. This might be due to the slippage synthesis [32–34] on the *psbA2* promoter, where the segment of AGUU transcribed from +1 to +4 slips back to the agtt template sequence from −4 to −1, resulting in an addition of 4 base extra sequence at the 5′-end (Figure 4(a)). Again, the *Syn* enzyme does not catalyze such a misincorporation.

### 3.5. Sensitivity to Mg^2+^ Ion of *Syn* RNAP

**Figure 5:**
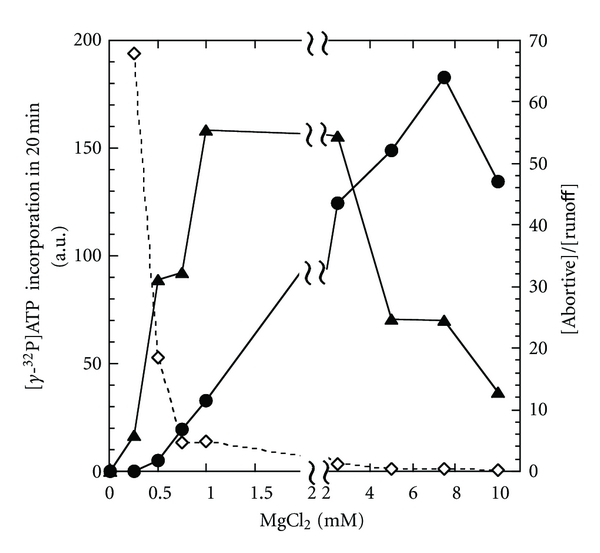
Abortive and runoff transcriptions by *Syn *RNAP at the *psbA2 *promoter in the different concentration of Mg^2+^. The levels of abortive synthesis of 3 to 11-mer (▲) and the runoff synthesis (●) in 20 min in a single-round are plotted against the concentrations of MgCl_2_. The ratio of the levels (*◊*) is also plotted.

The Mg^2+^ concentration in chloroplast is observed to be changed at dark and at light [35]. In cyanobacteria, the gene expressions are globally repressed at dark, including the *psbA2* expression, [36]. To test the possibility that transcription is directly controlled by Mg^2+^ concentration, we examined the sensitivity of *Syn* RNAP to the concentration (Figure 5). According to the increasing Mg^2+^ concentration from 0.5 to 2.5 mM, the level of runoff synthesis was increased by 25-fold, while the ratio of abortive synthesis to runoff synthesis was decreased to 1/15. In other words, it is possible to control RNAP to produce mainly abortive transcripts at a concentration and mainly mature transcripts in another concentration. However, such control would satisfy several quantitative conditions on the Mg^2+^ concentration. 

### 3.6. *Syn* RNAP Shows Higher Fidelity in Elongation Than the *E. coli* Enzyme

**Figure 6:**
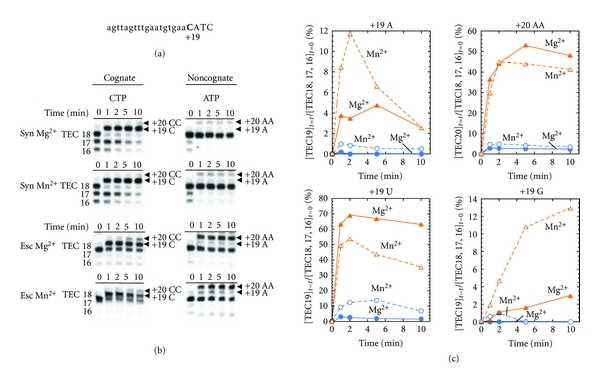
Incorporations of the cognate or noncognate NMP by the *Syn *and *E. coli *TEC18s in the presence of 10 mM Mg^2+^ or 1 mM Mn^2+^. (a) The sequence of the nontemplate strand from +1 to +22. Position +19 and the 18-mer transcript are shown in the bold letter and lower case letters, respectively. (b) The products of TEC18 synthesized in the presence of the cognate CTP and the noncognate ATP (0.5 mM each). Reactions were performed in the presence of 10 mM MgCl_2_ (upper) or 1 mM MnCl_2_ (lower). The lengths of transcripts are indicated on the both margins. The incorporated nucleotides are also indicated by the arrowheads in the right margin. (c) The amount of transcript involving misincorporation was shown in the percent of the initial amount of TECs. The TECs of the *Syn *(blue circle) and *E. coli *(*Esc*, orange triangle) were incubated with 0.5 mM NTP for the indicated times in the presence of 10 mM MgCl_2_ (filed symbols) or 1 mM MnCl_2_ (open symbols).

The results above described show that the *Syn* RNAP is slower and more accurate in the whole elongation than the *E. coli* RNAP. We then examined the elongation at the resolution of one nucleotide and arbitrarily selected the elongation at +18. By using a *psbA2* DNA with cytosine replaced as shown in Figure 6(a), we have prepared TEC18s by the *Syn* and *E. coli* enzymes. The elongations of *Syn* and *E. coli* TEC18s were carried out for 10 min with CTP, the cognate substrate, or ATP, a noncognate substrate, in the presence of 10 mM Mg^2+^ or 1 mM Mn^2+^ (Figure 6(b)). Similarly to TEC9s, a part of the transcript of the *Syn* TEC18 was shortened during the preparation, as shown in the lanes at time zero in Figure 6(b), probably due to hydrolysis or pyrophosphorolysis in the absence of NTPs. However, in contrast to TEC9, the shortening was not distinct for the* E. coli* TEC18, demonstrating that the *Syn* enzyme tends to shorten the retaining transcript more than the *E. coli* enzyme at Position +18.

 In the presence of the cognate CTP, the *Syn* and *E. coli* TEC18s incorporated CMP at Position +19 within 1 min and then slowly misincorporated CMP at Position +20 (Figure 6(b)). The level of the CMP misincorporation at 10 min was lower for the *Syn* TEC18 than for the *E. coli*, being consistent with the more accurate whole elongation by the *Syn* enzyme than by the *E. coli* enzyme. In the presence of the noncognate ATP, both the TEC18s incorporated two AMP molecules, the misincorporation at Position +19 and +20, and again the *Syn* enzyme was more accurate. The level of the successive two AMP incorporations was by an order of magnitude higher for the *E. coli* TEC18 than for the *Syn* one (Figure 6(c)). The *Syn* TEC18 showed higher fidelity than the *E. coli* TEC irrespective of the coexisting divalent cations, also for misincorporating UMP and GMP (Figure 5(c)). Because this misincorporation may be interpreted as the misincorporation at +19 with the substrate at +20, this misincorporation could be due to the misalignment mechanism [37, 38]. 

 In conclusion, the *Syn* enzyme showed higher fidelity than the *E. coli* one, both in initiation and in elongation, higher shortening activity, and slower rates of elongation. For most sets of substrates and enzymes, misincorporations were enhanced by the replacement of Mg^2+^ with Mn^2+^ except for the misincorporation by the *E. coli* TEC of AMP at +20 (Figure 6(c)).

## 4. Discussion

As shown in Figure 1(b), cyanobacterial RNAPs have large inserted domains in the G region of the *β*′ subunit [14]. In yeast RNAP II, the lack of Rpb9, which can be considered to be the counterpart of a part of the inserted domain of the bacterial *β*′ subunit, makes elongation faster and fidelity lower [4]. Our results share a similar feature: *E. coli* RNAP bearing smaller insertion elongates faster with lower fidelity than the cyanobacterial enzyme bearing a larger insertion. Therefore, the size of the inserted domain could be a determinant of the rate of elongation and the fidelity of an RNAP.

The holoenzyme containing the primary *σ* factor occupies less than 0.2% of the soluble proteins in cyanobacteria, and the content is at least 3 to 10-fold smaller than that in *E. coli *[24, 39, 40]. This is also consistent with the slower rates of elongation by cyanobacterial RNAP. As proved in *E. coli*, the release of sigma is a time-dependent event costing several seconds [41, 42], but elongation of several kilo base takes several minutes at least. Therefore, slower elongation requires less *σ* factor. In addition, the less formation of moribund complex also decreases the requirement of *σ* factor, because the complex contains the factor.

In *E. coli*, moribund complex blocks a promoter at the cost of abortive transcripts which are elongated at a rate of orders of magnitude smaller than the productive transcripts [30]. Therefore, repression by forming moribund complex at a promoter is considered to be less wasteful than abortion of long transcripts by elongation pause or immature termination (Figure  S3). The Gre factors enable the conversion of moribund complex into productive complex [31] to eliminate the repression by moribund complex [43]. The factors finely tune the levels of some proteins which are important to the growth in rich nutrient conditions [7, 44] although the factors are not essential. For example, the transcriptional level of *atp *operon encoding FoF1ATPase, the main generator of ATP, is reduced to one fourth in the disruptant of the *gre *genes [7]. Therefore, the Gre factors allow *E. coli* to make a quick uptake of nutrients at the moderate cost of abortive synthesis.

The two cyanobacteria examined in this study, as well as most cyanobacteria, grow in poorer nutrient conditions than *E. coli*. They may not be allowed to use the moderate cost as *E. coli* does with its Gre factors, and this might be the reason why they exceptionally lack the genes orthologous to *greA *which is widely conserved in eubacteria. Consistently, their RNAPs elongate transcripts slower and form less moribund complex, making the Gre factors insignificant. 

If the discussion above is the case, why is the branched pathway preserved in the cyanobacterial RNAP? There could be two answers. The first is that the mechanism is inevitably accompanied with the Brownian ratchet mechanism of RNAP elongation [45]. The other is that the cyanobacterial RNAP may use moribund complex only in limited conditions such as low Mg^2+^ concentrations. The ratio of abortive to runoff transcripts was 50 or more at a Mg^2+^ concentration lower than 0.5 mM (Figure 5), which is large enough for the repression by moribund complex to be significant. 

The concentration of Mg^2+^ in a stroma of chloroplast has been measured to change from 0.5 mM at dark to 2 mM at light [35]. This increase by illumination is due to the uptake of Mg^2+^ from the inner region of the thylakoid membrane, and electrically compensated by the antitransport of proton from a stroma due to photosynthesis [46, 47]. The cytosolic pH in cyanobacteria has been measured to change as in the stroma between light and dark [48, 49]. In contrast to Mg^2+^, little is known about the variation of Mn^2+^ concentration in cyanobacterial cells. Although a measured value of the concentration of Mg^2+^ or Mn^2+^ *in vivo* cannot be directly correlated to its concentration effect on *in vitro* transcription (Figure  S4), it is an important clue in comparative studies of RNAPs.

## Supplementary Material

The assignment of abortive transcripts are often confused by the presence of cleaved fragments of the run-off transcript. The cleavage occurs during the extraction with oxidized phenol but the fragments are distinguished by the retention of 3'-phosphate as described in Figure S2.
