# Supplementary material for: Comparative Study of Cyanobacterial and E. coli RNA Polymerases: Misincorporation, Abortive Transcription, and Dependence on Divalent Cations
Source: Genet Res Int. 2011 Oct 12;2011:572689. doi: 10.4061/2011/572689 (PMC3335489; doi:10.4061/2011/572689)
Supplement: Supplementary file 1 — The assignment of abortive transcripts are often confused by the presence of cleaved fragments of the run-off transcript. The cleavage occurs during the extraction with oxidized phenol but the fragments are distinguished by the retention of 3'-phosphate as described in Figure S2. [file 572689.f1.pdf]

Supplementary Figures

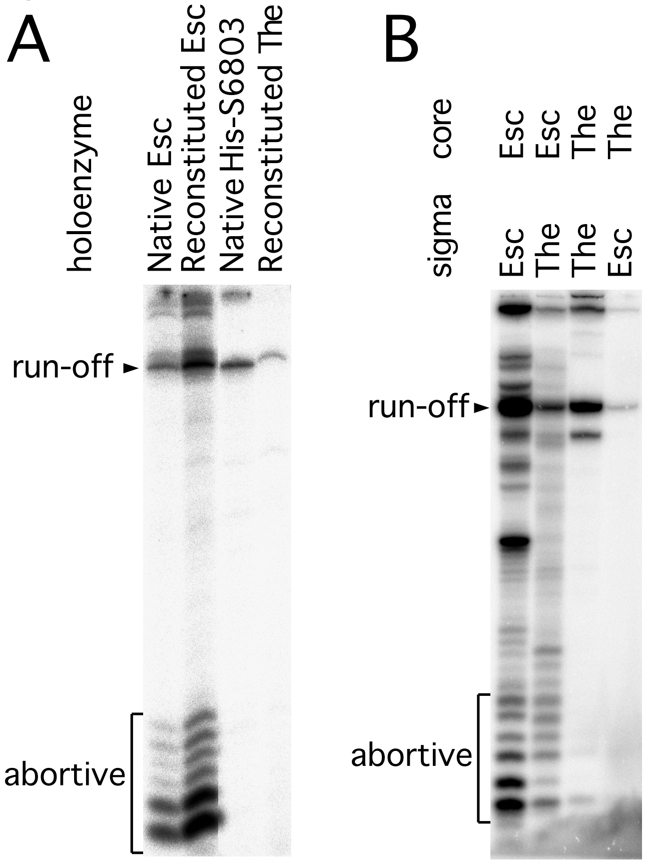

Fig. S1. Effects of reconstitution on abortive synthesis. A. Multi-round transcription from the *psbA2* promoter was carried out with [γ-^{32}P]ATP by the native and the reconstituted *E. coli* holoenzyme (Esc) purified as described [17], the histidine-tagged holoenzyme purified with Ni-affinity chromatography from *Synechocystis* sp. PCC 6803 (His-S6803) (lane 3) [51], and the reconstituted holoenzyme (R) of *The* (lane 4). **B.** Multi-round transcription was carried out with 1 μM of [α-^{32}P]UTP and 100 μM each of the other NTPs from the *rrnA* promoter by *E. coli* RNAP (lane 1) and *The* RNAP (lane 3) and their chimeric enzymes (lanes 2 and 4). The template DNA was the fragment of *rrnA* from -117 to +99 with +1 as the start site. It was prepared by PCR using the genomic DNA of *Synechocystis sp.* PCC 6803, and purified by PAGE. In the both panels, abortive and run-off transcripts are indicated by brackets and an arrowhead, respectively.

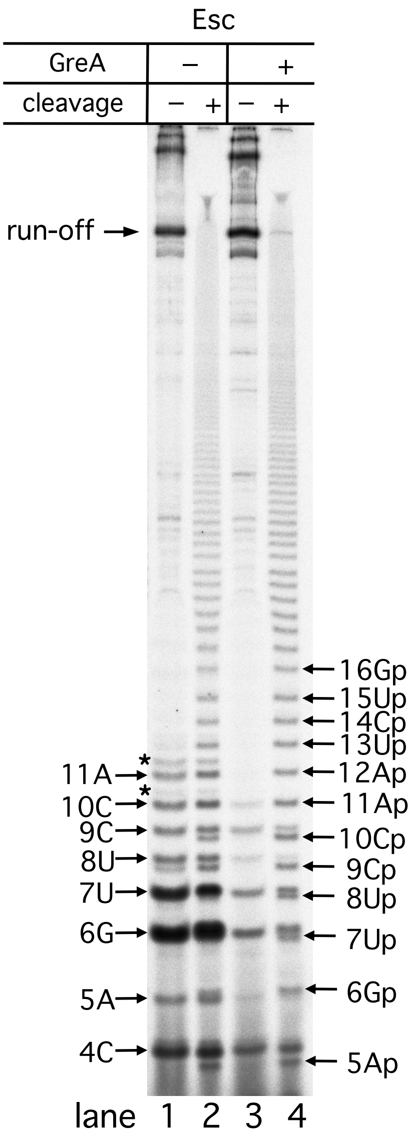

Fig. S2. Chemical cleavage of transcripts.

In Fig. 2B and 2C enbedded within the text, there were faint bands of various lengths such as a sequence ladder in the lanes with significant amounts of run-off transcripts. These bands are due to the cleavage occurring in extraction with the chloroform/ isoamylalcohol mixture with oxidized phenol.

Multi-round transcription from the *psbA2* promoter was carried out by the *E. coli* enzyme with [γ-^{32} P]ATP. The cleaved transcripts shown in lanes 2 and 4 retain 3'-phosphate and thus migrate faster than the uncleaved transcripts shown in lanes 1 and 3. The lengths of the cleaved and uncleaved transcripts are shown in the left and the right margin, respectively. The transcripts shown in lane 3 and 4 was obtained in the presence of GreA which decreases abortive synthesis.

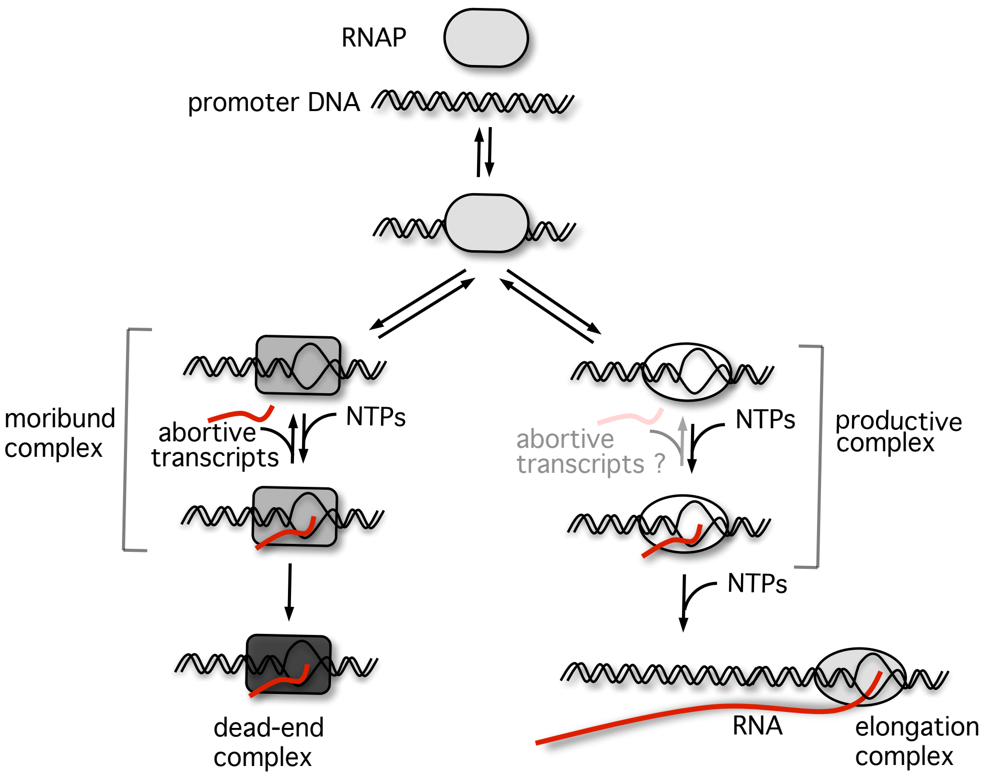

Fig. S3. The branched pathway of initiation. The initiation pathway iinvolves two branches: one leads to a productive complex and the other to a moribund complex [29]. RNAP holoenzyme and DNA carrying a promoter are, respectively, displayed as grey ovals and double helix. The moribund complex is a major source of abortive transcripts, but the absence of abortive synthesis in the productive branch has not been established. The fraction and reversibility in these branches is dependent on promoter and other factors such as Gre factors.

In *E. coli*, GreA and GreB tend to decrease abortive synthesis and to increase the full-length RNA synthesis by decreasing a fraction of the moribund complex. They increase the reversibility between moribund and productive complexes, and thus increase the flux through productive complex that is rapidly converted into elongation complex [44], while moribund complex is slowly converted into dead-end complex. The Gre factors are shown to increase the resistance against Mn^{2+} by unknown mechanism [7]. In cyanobacteria, no genes orthologous to *E. coli gre* have been found, although the intracellular concentration of Mn^{2+} is considered to be higher.

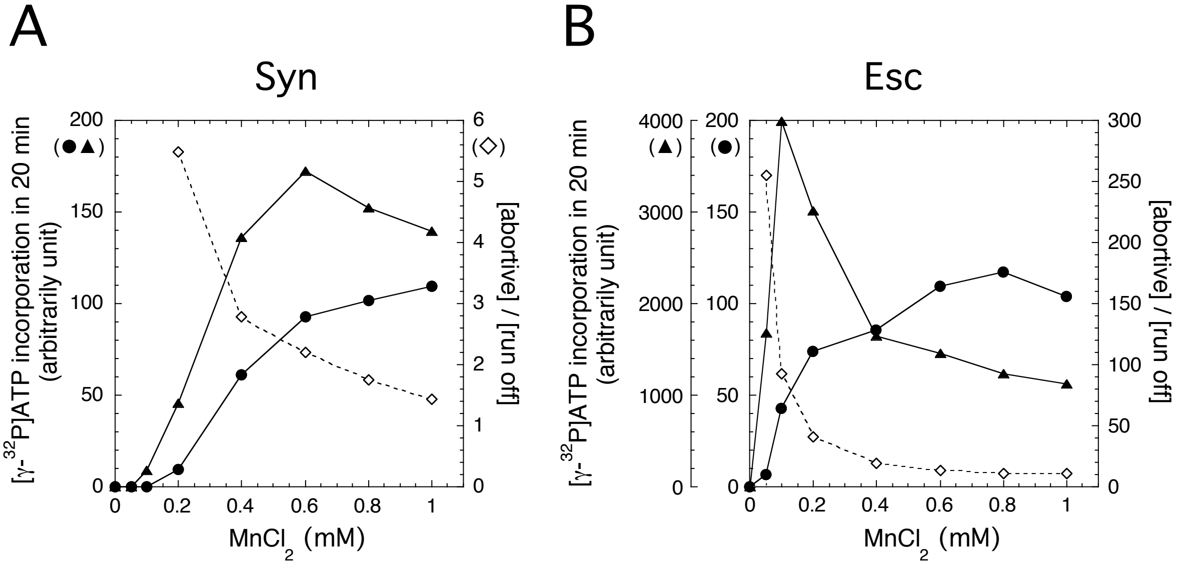

Fig. S4. Abortive and run-off transcriptions by *Syn* and *E. coli* RNAP at various concentrations of Mn^{2+}. For comparison of the effects of Mn^{2+}on initiation by *Sco* **(A)** and *E. coli* RNAPs **(B)**, a single-round transcription was carried out from the *psbA2* promoter at 1 mM or less concentrations, which are possibly close to physiological conditions [6]. The initiation of the run-off transcript (●) by the *Sco* enzyme reached maximum at 0.6 mM, while that by the *E. coli* enzyme was at 0.2 mM. In other words, the *Sco* enzyme is less sensitive to Mn^{2+} than the *E. coli* enzyme. A more prominent difference between these enzymes was found to be in the ratio of abortive synthesis to the run-off synthesis (◇): more than an order of magnitude difference throughout these ionic concentrations. The amounts of major abortive products, 3 to 11-mer (▲), and the run-off transcription, 20 min in a single-round condition, are plotted against the concentrations of $MnCl_2$.
